# The impact of real-time whole genome sequencing in controlling healthcare-associated SARS-CoV-2 outbreaks

**DOI:** 10.1093/infdis/jiab483

**Published:** 2021-09-23

**Authors:** Rodric V Francis, Harriet Billam, Mitch Clarke, Carl Yates, Theocharis Tsoleridis, Louise Berry, Nikunj Mahida, William L Irving, Christopher Moore, Nadine Holmes, Jonathan Ball, Matthew Loose, C Patrick McClure

**Affiliations:** 1 Department of Clinical Microbiology, Nottingham University Hospitals NHS Trust, Nottingham, UK; 2 School of Life Sciences, University of Nottingham, Nottingham, UK; 3 NIHR Nottingham Biomedical Research Centre, University of Nottingham, Nottingham, UK; 4 Wolfson Centre for Emerging Virus Research, University of Nottingham, Nottingham, , UK

**Keywords:** Whole genome sequencing: COVID-19, SARS-CoV-2, genetic epidemiology, nosocomial transmission, infection control, outbreak, cluster, virus

## Abstract

Nosocomial SARS-CoV-2 infections have severely affected bed capacity and patient flow. We utilised whole genome sequencing (WGS) to identify outbreaks and focus infection control resources and intervention during the UK’s second pandemic wave in late 2020. Phylogenetic analysis of WGS and epidemiological data pinpointed an initial transmission event to an admission ward, with immediate prior community infection linkage documented. High incidence of asymptomatic staff infection with genetically identical viral sequences was also observed, which may have contributed to the propagation of the outbreak. WGS allowed timely nosocomial transmission intervention measures, including admissions ward point of care testing and introduction of portable HEPA14 filters. Conversely WGS excluded nosocomial transmission in two instances with temporospatial linkage, conserving time and resources. In summary, WGS significantly enhanced understanding of SARS-CoV-2 clusters in a hospital setting, both identifying high risk areas and conversely validating existing control measures in other units, maintaining clinical service overall.

### Keywords

Whole genome sequencing: COVID-19; SARS-CoV-2; genetic epidemiology; nosocomial transmission; infection control; outbreak; cluster; virus

## Background

Severe acute respiratory syndrome coronavirus-2 (SARS-CoV-2) has become a global concern since being first reported in Wuhan, China in December 2019[1]. As of 10$^{th}$ May 2021, the UK has reported 4,437,217 cases with 127,609 deaths[2] and 463,820 hospital admissions. The clinical spectrum of COVID-19 infection is wide, ranging from asymptomatic infection to severe viral pneumonia leading to death[3, 4]. SARS-CoV-2 is highly transmissible by droplet and indirect contact [5]. Viral load in asymptomatic patients may be similar to those who are symptomatic[6]. The sensitivity of COVID-19 testing ranges from 71-98%[7]. This combination of factors poses a significant challenge in infection control.

Nosocomial transmission has been widely reported[8-14]. An early report from Wuhan showed that of 138 hospitalised COVID-19 cases, 12% were identified as being admitted for other reasons and presumed to have acquired the infection in hospital[14]. Transmission between hospital staff has also been demonstrated[13]. Retrospective analysis in a London teaching hospital showed nosocomial COVID-19 had a case fatality rate of 36%[11].

At Nottingham University Hospitals (NUH) Trust, United Kingdom, the Infection Prevention and Control team (IPCT) along with Clinical Microbiology colleagues have hitherto solely relied on epidemiological data to track outbreaks of viral pathogens [15]. Previously whole genome sequencing (WGS) has been used retrospectively to prove/disprove results of outbreak investigations [16]. Coronavirus Disease 2019 (COVID-19) Genomics UK Consortium (COG-UK) was set up in March 2020 to drive WGS nationally[17], and over 450,000 viral genomes have been sequenced (as of 10$^{th}$ May 2021). This is the first pandemic where WGS technology has been widely accessible in a clinically relevant timeframe, allowing exploration of its utility in a range of clinical scenarios [18-21].

We present a series of epidemiologically linked hospital clusters where WGS of SARS-CoV-2 isolates has directly affected real time outbreak management.

## Methods

All patients were tested for SARS-CoV-2 infection on admission to NUH Trust, irrespective of symptomatology. All samples underwent RT-PCR through one of four different molecular platforms (Supplementary information). Use of surplus nucleic acid derived from routine diagnostics and associated patient data was approved through the COG-UK consortium by the PHE Research Ethics and Governance Group (R&D NR0195).

At the time of analysis, all inpatients with negative admission tests were re-tested every 7 days. Patients who tested negative on admission but fulfilled clinical criteria for COVID-19 infection were managed similarly to positive patients.

Positive patients were moved to a COVID-19 ward where they were isolated in a side room or cohorted with other positive patients in a bay. Patients negative for COVID-19 were moved to a non-COVID-19 ward. If a bed was not available immediately, patients were isolated or cohorted appropriately on the admissions ward until a bed became available. Type IIR surgical mask, plastic apron and gloves were used as standard personal protective equipment (PPE) on all wards when caring for patients, with enhanced PPE when aerosol generating procedures were carried out. Staff were advised to always wear Type IIR surgical masks when inside the hospital which should only have been removed when consuming food or drink.

Symptomatic staff testing was also undertaken. During outbreaks, asymptomatic screening of all healthcare workers who worked in the particular area of concern was also undertaken. Until November 2020, there was no routine asymptomatic healthcare worker testing. The average turnaround time to SARS-CoV-2 results was 11.5 hours from when the swab was received in the laboratory. Results were alerted to the clinical team electronically as soon they were available.

Samples with a positive RT-PCR result (with cycle threshold of <35) and suggestive epidemiological linkage underwent WGS. For routine surveillance of viral sequences not flagged by epidemiological linkage, samples testing positive on the Altona platform with a cycle threshold value of <30 were selected for sequencing. WGS was performed using the ARTIC amplicon sequencing protocol on Oxford Nanopore GridION sequencers to enable rapid turnaround [22] (Supplementary information). V3 primers (as described at https://github.com/artic-network/primer-schemes/tree/master/nCoV-2019/V3) were used for all samples. Samples were typically sequenced and analysed within 72 hours of collection, with analysis using the artic pipeline as described in [22]. Daily communication between the IPC team and sequencing laboratory identified all new positives within the last 24 hours and enabled randomised sample selection as well as targeted sample collection for further investigation. All samples in this study are available through GISAID (if coverage >90%), from COG-UK (https://www.cogconsortium.uk/tools-analysis/public-data-analysis-2/) or via ENA. A table of COG-UK IDs used in this study is provided (see Supplementary Table 1). Samples were analysed using the CIVET tool (version 2 - https://github.com/artic-network/civet ) in real time. Further analysis is as described in the supplementary text.

## Results

### Cluster 1

Patients with a diagnosis of a respiratory condition were admitted from both the Emergency Department (ED), as well as directly from the community to a triage respiratory admissions ward, Ward X. A diagnosis of COVID-19 infection was based on a laboratory result, or more rarely on clinical criteria despite negative laboratory testing. Based on this, patients were transferred to a COVID-19 or a non-COVID-19 ward as appropriate.

Cluster 1 took place on Ward Y; an adult single sex non-COVID-19 respiratory ward, consisting of five bays with six beds in each and four side rooms. A timeline for this outbreak is shown in Figure 1.

Patient A was admitted to Ward X with breathlessness secondary to a pleural effusion. This marks Day 0 for the purposes of this cluster. The admission SARS-CoV-2 swab was negative. The patient was transferred to Ward Y three days later. On day 8 of admission, the patient developed a fever and worsening hypoxia and a repeat swab detected SARS-CoV-2. The patient was then isolated in a side room before being transferred to a COVID-19 ward.

Patient B was also admitted to Ward X on Day 0 and had a negative result. They were discharged from hospital on Day 4 but subsequently readmitted 2 days later to Ward X, when their admission test detected SARS-CoV-2. WGS results identified Patient A and Patient B's SARS-CoV-2 genomes as identical.

Patient C was admitted to Ward X on Day 1 with a SARS-CoV-2 induced exacerbation of chronic obstructive pulmonary disease (COPD). Subsequent WGS surveillance identified Patient C to be infected with a genetically identical SARS-CoV-2 virus to Patients A and B (Figure 2). Detailed analysis of the outbreak timeline (Figure 1) in conjunction with the WGS results identified a probable transmission point. All three patients had been located in a bay on Ward X for just 6 hours. This suggested Patient C as the likely index case. This observation is further supported by the later identification of 5 community samples (identified as direct close contacts of Patient C) at the base of this branch (Figures 3a and 3b) Patient C differs from these linked community samples by one single SNP, G12052T, which defines all subsequent members of this cluster and is discussed in more detail below. These genomes are all members of Pango lineage B.1.177.57. This lineage was first reported 2020-06-02, with 3,343 samples reported to date (https://cov-lineages.org/lineage.html?lineage=B.1.177.57) [23]

Patient D was admitted to Ward X on Day 5 with pulmonary oedema. They tested negative for SARS-CoV-2 and were moved to Ward Y into an open bay. On Day 12 their weekly screening swab flagged positive for SARS-CoV-2. They were promptly moved to a COVID-19 ward. Although only a partial genome sequence was obtained, 14/18 SNPs identified in the viral genome of Patient D matched those of Patients A, B and C with no unique SNPs in this sample.

An outbreak was declared as Patient D had not been a contact of Patient A, B or C. Ward Y was closed for new admissions and asymptomatic screening of patients and staff was undertaken. WGS identified that viruses from Patients E, H and M as well as two staff members (Staff A and E) shared identical SNPs with the original cluster (Supplementary information). In total, 8 staff on Ward Y were observed to carry this lineage (3 or fewer differences in SNPs) and were predominantly asymptomatic; staff B & D carried other lineages. This may explain the further spread within Ward Y with no clear contact between the patients.

Five additional patients on Ward Y (Patients J, K, L, N and O) tested positive during Days 20-29. WGS revealed these patients shared all 18 SNPs with the original cluster, but had an additional 2 SNPs (C16883A, C28087T). These SNPs were also found in three healthcare workers from Ward Y (Staff G, H and I). WGS further identified 5 patients on Ward J and 6 patients on Ward K with near identical strains (Supplementary information).

Phylogenetic analysis showed that all Cluster 1 isolates formed a distinct clade (Figure 3). The clade also contained the sub-cluster of 5 community acquired isolates (Community A-E) which were antecedent to all the hospital acquired ones, suggesting this as the route of transmission from community to hospital (Figure 3b). The second sub-cluster comprised 10 Ward Y sequences (Patient A, B, C, E, F, H and Staff A, E, L, M). There were 2 descendant sub-clusters; the first was comprised entirely of 8 Ward K isolates (Patient U, V, W, X, Y, Z, Aa and Ab), indicating transmission from Ward Y to Ward K. The second one included a mixture of isolates from patients and staff of Ward Y (9 in total) as well as patients of Ward J (5 in total), suggesting multiple transmission events between Wards Y and J.

Overall, the epidemiological data combined with WGS and phylogenetic analysis suggest that nosocomial transmission from a single patient led to clusters of patients and staff positive for SARS-CoV-2 B.1.177.57 lineage on three wards in the hospital. The primary ward, Ward Y, had 13 patients and 11 staff members positive for SARS-CoV-2. Ward J had 5 patients and Ward K had 8 patients infected as a consequence of this outbreak. All 3 wards experienced associated mortality in line with previous studies of hospital acquired SARS-CoV-2 infection (data not shown, [11, 24, 25]).

The lineage for this strain, B.1.177.57, is a child lineage of B.1.177, the strain most prevalent in the UK at the time [26]. B.1.177 was at least 10-fold more prevalent than any other strain in the East Midlands area as of December 2020, representing approximately 70% of samples identified to date in the area (Supplementary Table 2). More recently, B.1.1.7 has displaced this lineage in the East Midlands area[27]. Although formally impossible to exclude multiple introductions of B.1.177.57 into the hospital, the lineage defining SNP, G12052T, is extremely informative. This SNP has occurred independently in multiple lineages, with 103 individual sequences containing this SNP reported in the UK as of July 2021. However, within B.1.177.57 lineage sequences, this SNP only occurs 45 times. Of these, 34 are within the cluster reported here and the first identified occurrence of this SNP anywhere in this lineage is Patient C, the likely index case. Of the remaining 11 samples, 1 was a bank staff member later identified to have been present on this ward and 2 were patients with known contacts with the outbreak from within the hospital but not part of the initial investigation (Supplementary Table 1, Figure 3b). A further 8 genomes were sequenced in the community as part of the efforts of COG-UK, all collected at least 1 month after the initial outbreak within the hospital (Supplementary Table 1, Figure 3b). Although impossible to prove, it is likely that some of these cases are from health care workers from these wards who were tested for COVID-19 in the community. Thus the most parsimonious explanation is that this outbreak originated with Patient C.

**Cluster 2**

Cluster 2 took place in a renal dialysis unit. Patients in this area were tested for SARS-CoV-2 if they were symptomatic or had been in recent contact with a confirmed positive patient. Patients were allocated an area in the dialysis unit based on their COVID-19 status.

Two cases of SARS-CoV-2 infection were identified in haemodialysis patients attending the same unit. The first, asymptomatic, patient (Patient A2) was screened and tested positive for SARS-CoV-2. Two days later, the second case (Patient B2) attended and tested positive, requiring admission to hospital with hypoxia. An outbreak was declared and screening was performed on 26 patient contacts, all asymptomatic. A third case (Patient C2) was identified from screening which suggested an evolving outbreak. This would have impacted functioning of the dialysis unit, leading to a major operational issue for the Trust. WGS data indicated that the isolates from the first 2 cases were identical but the third case was different (Figure 4). The Pango lineage of the shared cases was B.1.1.37, whilst the lineage of the individual case was the more common B.1.177 (at the time). B.1.1.37, although similar to that of Cluster 1, is clearly distinct (Figure 3a). The IPCT refocused their investigation on the two identical cases. Patient pathways outside the dialysis unit were reviewed. The only identified connection between them was that both patients travelled together regularly sharing the same ambulance transport to the dialysis unit. A third patient who was also in the ambulance car was negative on both initial screening and on re-testing 7 days later. The driver of the ambulance car was not tested and remained asymptomatic.

A review of the processes around transporting patients to the dialysis unit was undertaken. There was additional focus on appropriate mask usage in the car (type IIR surgical face mask). All patients when interviewed stated that the appropriate mask had been worn at all times in the car. Additional patient education of all patients travelling to the dialysis unit was undertaken in response to this event.

**Cluster 3**

Cluster 3 took place on a paediatric general surgical ward, Ward D, which has 11 beds and 4 side rooms. Patients arrive here from a paediatric admissions ward following initial review.

The first case identified (Patient A3) was an infant who had a 58 day inpatient stay following surgical intervention for a prolapsed stoma. During this admission the patient had a negative swab result, and was discharged 8 days later. Nine days following discharge they were readmitted with a positive admission screen for SARS-CoV-2. The previous day a symptomatic staff member (Staff A3) who regularly worked on Ward D tested positive. The possibility of nosocomial transmission was considered, either patient to healthcare worker, healthcare worker to patient, or a third point source as yet undetected, therefore all patients on the ward were screened. This did not identify any further patient cases. No other healthcare workers reported any symptoms. There remained ongoing clinical concerns of a developing outbreak. Wider asymptomatic healthcare worker screening was being considered. Rapid WGS was undertaken, which showed that the patient's lineage (Patient A3 – B.1.177.16), was very different to the healthcare worker's strain (Staff A3 – B.1.36.17) (Figures 3a and 4). Hence an outbreak was excluded and the ward returned to its normal processes.

**Discussion**

The ability to use WGS data at scale to inform hospital outbreaks in close to real time has only been possible in the last decade [8, 28-30]. Most recently, it has become possible to routinely sequence samples within a 48-hour turnaround and feedback to clinical teams whilst an outbreak may be in progress. This has been facilitated by the co-ordinated work of the COG-UK consortium enabling distributed sequencing throughout the UK [17]. Here we present 3 clusters of SARS-CoV-2 infections which used this rapid feedback to directly inform active outbreak investigations, allowing the IPCT to focus resources on genetically linked outbreaks, which was of particular value during periods of high SARS-CoV-2 positivity.

We have been able to identify transmission events occurring in our admission areas using genomics to support the likely routes of transmission. In the case of Cluster 1, the uniquely occurring SNP in the context of the specific lineage under observation suggests an outbreak initiating within the hospital and potentially spreading into the community. Mortality within this cluster was as would be expected at this period of the outbreak on these type of wards [11, 24, 25].

From the examples presented, the severe impact of a hospital outbreak is evident. Cluster 1 resulted in numerous infections affecting multiple wards. The ongoing data stream provided by rapid WGS supplemented the IPCT epidemiological investigation and allowed identification of additional patients involved in the outbreak. As evidenced by the SNP profiles and phylogeny, patients in Cluster 1 with no direct contact still shared the same lineage. Given that many of the staff who tested positive were asymptomatic, they would have continued working until screening was performed as part of the outbreak management process.

Although the strain B.1.177 was abundant in the UK during the study period, accounting for nearly 75% of all cases in the region [27], this cluster shared a unique SNP specific to this outbreak. In the course of our investigations, we also identified five samples linked to the index patient that had been diagnosed in the community. These individuals provide the community context by which this lineage entered the hospital but do not contain the SNP unique to the outbreak. There is no evidence at this time that this unique SNP confers any specific phenotype on the lineage. Coupled with the extensive epidemiological data supporting links between these patients and staff, Cluster 1 is a significant hospital outbreak. The application of WGS at the onset of this outbreak enabled the rapid identification of nosocomial transmission and linkage to other wards.

Use of WGS allowed us to identify that transmission of SARS-CoV-2 was occurring in our respiratory admissions area, Ward X. Recognising this allowed us to alter practice quickly, introducing additional control measures. Following this outbreak, a Cepheid GeneXpert ® Xpress has been placed on Ward X as well as in the Emergency Department with support of the Point of Care Testing team to further reduce delays caused by transportation of samples[31]. This helps prompt isolation or cohorting of COVID-19 patients, reducing the risk of nosocomial spread to patients who are admitted due to another illness [32]. Air Sentry® HEPA14 air filters have also been deployed onto the admission wards as well as base wards where there has been increased nosocomial transmission [33]. Cluster 1 also highlighted that patients moving between wards can perpetuate ongoing transmission as occurred with Patient K (Supplementary Figure 1). This cluster also emphasises the importance of future design planning of admission areas to ensure adequate isolation and effective ventilation is available[34].

In Cluster 2, the WGS results and epidemiological link between the first two cases, strongly suggested that there had been one transmission event likely to have occurred whilst the patients shared transport as no other cases sharing the same WGS were identified in the dialysis unit. This helped close an outbreak investigation quickly, providing assurance that the infection prevention and processes applied within the unit were working well. To date there have been no other transmissions linked to patient transport following additional patient education and emphasis on mask wearing and hand hygiene during patient ambulance transport. This occurrence is similar to that seen in other health care contexts earlier in the pandemic [8].

The same applies to Cluster 3, where the clear-cut difference in the strains and weak epidemiological link within the hospital led to the conclusion that the viruses in both patient and staff member had likely been acquired in the community. Prior to WGS, the monitoring process associated with investigation of a potential outbreak would have required considerable time and resources. Cluster 1

and Cluster 2 developed around the same time. WGS results helped focus our efforts on Cluster 1 where nosocomial transmission occurred.

No further cases of epidemiologically or phylogenetically linked SARS-CoV-2 infection were identified in settings related to Cluster 2 and 3 in the weeks following this investigation, further supporting the interpretation of real-time WGS and clinical decisions actioned. In general, these two scenarios highlight the greater ability of WGS evidence to disprove transmission, as opposed to Cluster 1 where the potential for extraneous re-introduction of highly related or identical virus could not be completely eliminated but was not the most parsimonious interpretation.

In conclusion, real-time WGS in association with strong epidemiological evidence has proven to be highly useful in identifying and intervening in hospital SARS-CoV-2 outbreaks and maintaining continuity of service during periods of high viral prevalence, by identifying an outbreak in one of our respiratory admission areas, and disproving nosocomial transmission in 2 other settings despite classical epidemiological methods suggesting linkage.

There is a strong case to be made for continued routine surveillance of infection in local health care settings. Delivering WGS results within 48 hours enables direct response by the IPCT, although even faster turnaround time would have been beneficial in rapid tracing of Cluster 1. These rapid responses can only currently be achieved where sequencing is embedded as closely as possible within the clinical workflow. Implementation and dissemination of WGS in healthcare settings should therefore continue to be supported and strengthened both nationally and globally.

## Figure Legends

**Figure 1.** Cluster 1 outbreak timeline, showing patients (A to O), ward location and designation and SARS-CoV-2 RT-PCR testing in relation to time in days, with day 0 marking the admission of patients A & B to Ward X. The temporospatial window for the putative initial transmission event on Ward X, with patients A & B sharing a bay with patient C for 6 hours only on day 1, is highlighted by a red box. Temporal SARS-CoV-2 RT-PCR positivity and healthcare role is also presented for staff (A to L) who were linked by both classical and genomic epidemiology. All staff, with the exception of bank staff L, were based on Ward Y. WGS determined viral sequences in all patients and staff belonged to the same lineage with the exception of staff B & D (Supplementary Table 1). The colour scheme key denotes ward location and RT-PCR test results, with broken timelines indicating discharge and subsequent readmission of the patient.

**Figure 2.** Snip-it plot from the CIVET tool (https://github.com/artic-network/civet) showing 29 SNPs and genomic positions differing from the B.1.177 reference sequence for samples associated with Cluster 1. SNPs highlighted in yellow boxes are shared between all samples including those from the community surveillance. The G12052T SNP (highlighted in the red box) is shared between all samples taken from patients and staff within the hospital environment. N indicates positions in samples where no base was called and relative base position in the SARS-CoV-2 genome is indicated at the foot of the figure.

**Figure 3.** Phylogenetic relationship by maximum likelihood analysis of all whole genome SARS-CoV-2 sequences (29,574 bases) from described hospital clusters 1 (coloured in red, purple and brown for Wards Y, J and K respectively), 2 (blue) and 3 (green) and contemporary sequences from Nottingham, UK collected between 01/09/2020 and 30/10/2020 (panel A) and additional sequences sharing SNP G12052T in the B.1.177.57 lineage. Distinct subgrouping of cluster 2 and 3 sequences is supported by bootstrap values of 99.2 and 100 respectively for basal branches (not shown). A focused phylogenetic subtree of Cluster 1 is also presented in panel B, coloured as per panel A. Dashed lines indicate branches for community samples sequenced elsewhere without metadata available for further investigation; numbers above individual branches indicate SH-aLRT bootstrap support with values less than 70 not shown, and some values of 100 indicated by an asterisk for clarity. Sequences that did not meet the >95% genomic coverage criteria were excluded from the analysis. Sequences are identified by their COG-UK accession numbers. Branch lengths are drawn to a scale of nucleotide substitutions per site, with scale indicated.

**Figure 4.** Snip-it plot from the CIVET tool (https://github.com/artic-network/civet) showing SNPs and genomic positions differing between viral sequences from individuals involved in Cluster 2 & 3 and the ancestral Wuhan-Hu-1 lineage B reference sequence (MN908947.3) Relative base position in the SARS-CoV-2 genome and study sequence lineages are indicated at the foot and right of the plot respectively.

**Supplementary Figure 1.** Extended Cluster 1 outbreak patient timeline, showing patients (A to Ab), ward location and designation and SARS-CoV-2 RT-PCR testing in relation to time in days, with day 0 marking the admission of patients A & B to Ward X. The temporospatial windows for the putative transmission events are highlighted by red boxes. WGS determined viral sequences in all patients belonged to the same lineage (Supplementary Table 1). The colour scheme key denotes ward location and RT-PCR test results, with broken timelines indicating discharge and subsequent readmission of the patient.

Figure 1

Accepted

Figure 2

Accepted

Figure 3

A
Cluster 1
Cluster 3
Cluster 2
Cluster 2
Cluster 3
8.0E-5
B
9 Sequences
NOTT-1131CA
NOTT-1133C4
QEUH-9DE4AC
QEUH-9C9D8B
Community D (NOTT-113C9C)
Community A (NOTT-113582)
Community E (NOTT-11382F)
Community B (NOTT-113810)
Community C (NOTT-113CAB)
Staff C (NOTT-114BF8)
Patient F (NOTT-114448)
Patient U (NOTT-114F65)
Patient V (NOTT-115359)
Patient Z (NOTT-117C4D)
Patient Y (NOTT-11767C)
Patient X (NOTT-118A42)
Patient Ab (NOTT-117779)
Patient W (NOTT-11614F)
Patient Aa (NOTT-11768B)
Patient O (NOTT-116B6F)
Staff K (NOTT-1163D0)
Patient N (NOTT-11582D)
Patient P (NOTT-117D95)
Patient K (NOTT-1151AA)
Patient - Known Contact (NOTT-115131)
Patient Q (NOTT-118945)
Staff G (NOTT-114D5C)
Patient J (NOTT-1148D3)
Patient T (NOTT-1186D5)
Patient S (NOTT-118E28)
Staff I (NOTT-115298)
Staff (Bank) (NOTT-1153C2)
Patient R (NOTT-1196D4)
Patient L (NOTT-1151C8)
Untracked Community (QEUH-AF068F)
Patient G (NOTT-1148C4)
Patient - Known Contact (NOTT-11578A)
Untracked Community (QEUH-AD5592)
Staff E (NOTT-114B61)
Untracked Community (ALDP-C3B98D)
Untracked Community (QEUH-BF0D4D)
Untracked Community (CAMC-B3419C)
Untracked Community (MILK-ACD5CA)
Untracked Community (MILK-B38C6A)
Untracked Community (QEUH-B11B3D)
Patient C (NOTT-1134B2)
Patient B (NOTT-113B35)
Staff A (NOTT-114C04)
Patient H (NOTT-11493A)
Patient E (NOTT-114536)
Staff F (NOTT-114C40)
Staff L (NOTT-115632)
Patient A (NOTT-113AA1)
Patient M (NOTT-115104)
100
86.5
76.5
87.6
85.3
88.1
91.5
80.9
79.5
78.8
87.4
73.9
3.0E-5

Figure 4

## Supplementary Material

jiab483_suppl_Supplementary_Figure_S1

jiab483_suppl_Supplementary_Materials

jiab483_suppl_Supplementary_Table_S1

jiab483_suppl_Supplementary_Table_S2

