# Supplementary figures and images for: The Impact of Real-Time Whole-Genome Sequencing in Controlling Healthcare-Associated SARS-CoV-2 Outbreaks
Source: J Infect Dis. 2021 Sep 23;225(1):10–8. doi: 10.1093/infdis/jiab483 (PMC8522425; doi:10.1093/infdis/jiab483)

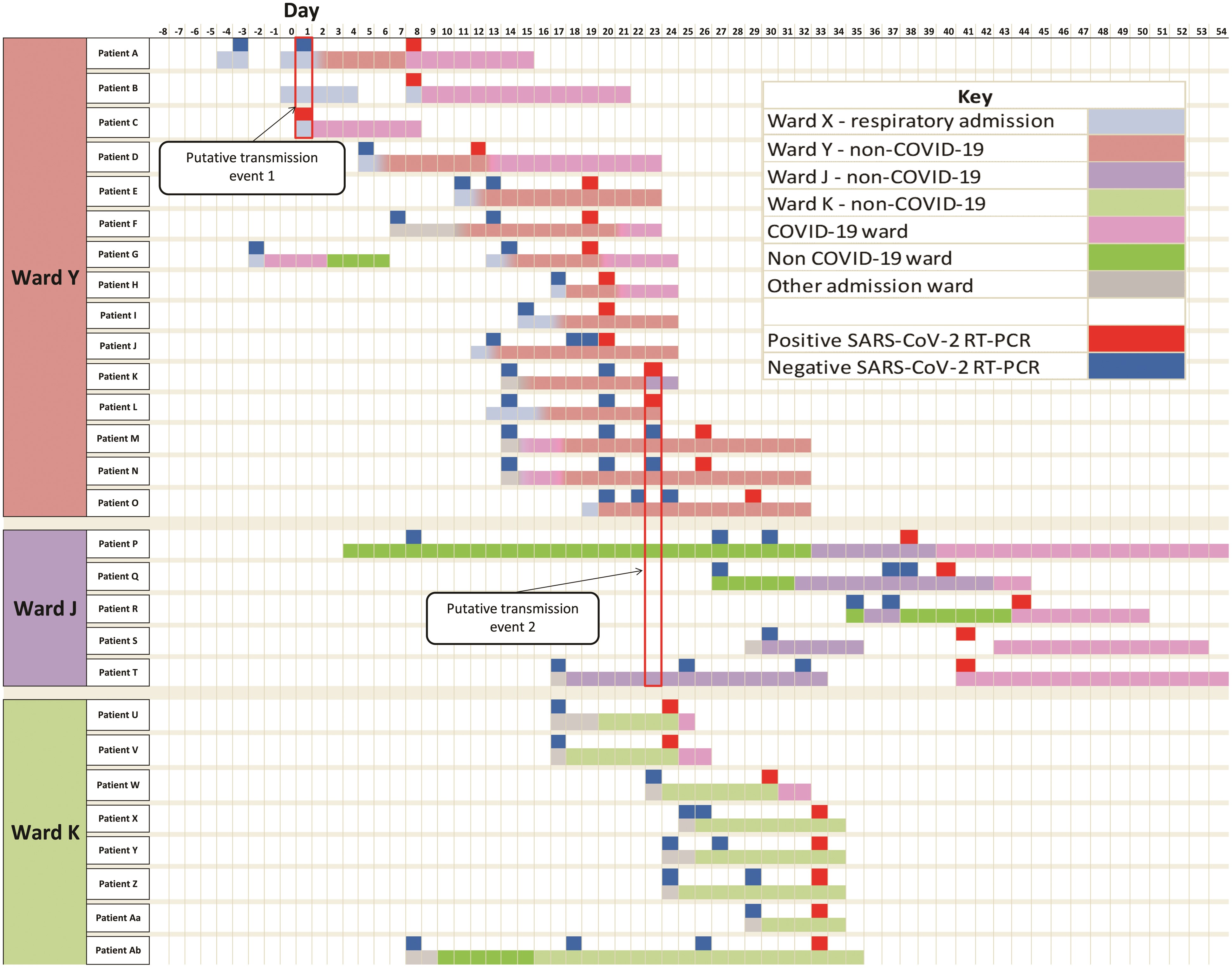

Supplement: jiab483_suppl_Supplementary_Figure_S1 [file jiab483_suppl_supplementary_figure_s1.jpeg]
