# Supplementary material for: The Impact of Real-Time Whole-Genome Sequencing in Controlling Healthcare-Associated SARS-CoV-2 Outbreaks
Source: J Infect Dis. 2021 Sep 23;225(1):10–8. doi: 10.1093/infdis/jiab483 (PMC8522425; doi:10.1093/infdis/jiab483)
Supplement: jiab483_suppl_Supplementary_Materials [file jiab483_suppl_supplementary_materials.docx]

**Supplementary information**

*SARS-CoV-2 RT-PCR*

Nasopharyngeal swabs were undertaken using Sigma swabs in Virocult® viral transport medium. Upon receipt samples were placed on one of the laboratory multiple work streams based on priority, location and time of day received. Criteria determining which platform was to be utilised for any individual sample were outlined in a Standard Operating Procedure (SOP) through the organisational structures and depended on which area of the hospital the sample was collected from, the need for expediency of the result, and availability of reagents. The platforms used are described below.

*Work stream 1*

Extraction is performed using three different platforms.

- AltoStar® Automation System AM16 (Altona Diagnostics) assay with purification performed using AltoStar® Purification Kit 1.5
- NucliSens easyMAG® extraction system
- Maxwell® RSC Instrument Purification.

Following extraction on one of the above methods, RT-PCR is performed on Bio-Rad CFX96™ Real-Time PCR assay detects SARS-CoV-2 *E* and *S* gene targets.

*Work stream 2*

The *m*2000 RealTi*me* System was used to perform the Abbott RealTi*me* SARS-CoV-2 assay. This system comprises a sample preparation unit *m*2000*sp*, and amplification and detection unit (Abbott *m*2000*rt)*. This is a dual target assay detecting RNA‐dependent RNA polymerase (*RdRp*) and nucleoprotein (*N*) genes.

*Work stream 3*

Cepheid GeneXpert Xpert® Xpress detecting molecular targets *E* and *N* proteins – used for rapid turnaround including emergency surgery or transplant patients.

*Work stream 4*

Novodiag® COVID-19. detecting molecular targets *orf1ab* and *N* proteins – also used for rapid turnaround

*Whole genome sequencing*

The resulting genomes from the ARTIC amplicon sequencing protocol were aligned and 5’ and 3’ ends were trimmed. The alignment included sequences collected from Nottingham between 01/09/2020 and 30/10/2020 with >95% coverage downloaded from COG-UK (889 sequences). The processing of the genomes was performed using the Geneious Prime 2019.0.4 software. Lineages were assigned to the genomes using the Pangolin tool [2]. A Maximum-Likelihood tree was generated to assess the evolutionary relationships between outbreak sample genomes and other genomes obtained from Nottingham and UK patients. The tree was generated with IQ-TREE2 [3] using the General time reversible model with empirical base frequencies and FreeRate (GTR+F+R2) model of evolution as suggested by the software’s model finder with 1000 SH-like approximate likelihood ratio test (SH-aLRT) [4]. The trees were annotated with Fig tree v1.4.4. Custom SNIPIT plots were generated using the SNIPIT tool (https://github.com/aineniamh/snipit).

**Additional information relating to WGS of Cluster 1**

Patient C has 1 SNP different to the community samples A to E, namely G12052T. Thus a set of 18 SNPs were identified in common in all subsequent individuals in Cluster 1 (see Figure 1 & 2). This cluster is from pangolin lineage B.1.177.57.

WGS identified that Patients E, H and M as well as two staff members (Staff A and E) shared identical SNPs with the original cluster. For one member of staff (Staff J) WGS only generated a partial genome, but again the SNPs that could be called (13/18) were identical to the main cluster. Patients F and G were separated by 1 SNP (C21575T) and two SNP’s (C16883A, T29047C) respectively. Although Patient I tested positive, WGS was unsuccessful for this patient (Supplementary Table 1).

Amongst the remaining staff on the ward, two staff harboured viruses which differed by 1 SNP (Staff F and H), two staff by 2 SNPs (Staff C, G and I) and 1 staff by 3 SNPs (Staff K). All these staff shared the core set of SNPs. Virus from one remaining staff member (Staff D) was determined to be of lineage B1.177.16 (Supplementary Table 1) and only shared 12/18 SNPs with this cluster with an additional 3 SNPs with respect to the reference strain giving a total of 9 nucleotides difference. As a consequence, this individual was not considered part of this cluster. Within the wider hospital staff community only two other staff members, Staff L and a further bank staff (Figure 1 and Supplementary Table 1), had a virus of the same lineage as the cluster and shared 18/18 SNPs.

WGS further identified 5 patients on Ward J and 8 patients on Ward K with near identical strains (See Supplementary Figure 1 and Figure 2). These samples held all SNPs in common with samples from Patients P, Q, R, S and T from Ward J. Patient P first tested positive on 26^th^ October and so predated the variants found on Ward Y. Ward J is a 20 bedded adult renal medicine ward with 4 side rooms and 4 bays with 4 beds in each bay. Patient K was moved from Ward Y to Ward J on the 11^th^ October (see Figure 1 and Supplementary Table 1) for 48 hours. During this time Patient T was also present on this ward. This identified a potential transmission event on Ward J. (see Supplementary Figure 1)

Another link was identified by WGS on Ward K, a 28 bedded care of the elderly ward with 4 side-rooms and 4 bays with 6 beds in each bay. Again, 8 patients (Patients U, V, W, X, Y, Z, Aa and Ab) were found to share all 18 SNPs with the original cluster (See Supplementary Figure 1 and Figure 2). All 8 patients shared an additional SNP (C7932T). Patients V, Y, Z and Ab each had an additional SNP suggesting further within-ward transmission. No staff members on Ward Y held this specific subset of SNPs.

1. Chappell JG, Tsoleridis T, Clark G, et al. Retrospective screening of routine respiratory samples revealed undetected community transmission and missed intervention opportunities for SARS-CoV-2 in the United Kingdom. medRxiv **2020**:2020.08.18.20174623.

2. Rambaut A, Holmes EC, O'Toole A, et al. A dynamic nomenclature proposal for SARS-CoV-2 lineages to assist genomic epidemiology. Nat Microbiol **2020**; 5:1403-7.

3. Minh BQ, Schmidt HA, Chernomor O, et al. IQ-TREE 2: New Models and Efficient Methods for Phylogenetic Inference in the Genomic Era. Mol Biol Evol **2020**; 37:1530-4.

4. Guindon S, Dufayard JF, Lefort V, Anisimova M, Hordijk W, Gascuel O. New algorithms and methods to estimate maximum-likelihood phylogenies: assessing the performance of PhyML 3.0. Syst Biol **2010**; 59:307-21.
