# Supplementary material for: The Impact of Real-Time Whole-Genome Sequencing in Controlling Healthcare-Associated SARS-CoV-2 Outbreaks
Source: J Infect Dis. 2021 Sep 23;225(1):10–8. doi: 10.1093/infdis/jiab483 (PMC8522425; doi:10.1093/infdis/jiab483)
Supplement: jiab483_suppl_Supplementary_Table_S1 [file jiab483_suppl_supplementary_table_s1.docx]

**Supplementary Table 1** Cluster sequence ID numbers and details

| Cluster | Location | Manuscript ID | COG-UK ID | GISAID Accession ID | Collection date | Pango lineage | Completeness ^a^ |
| --- | --- | --- | --- | --- | --- | --- | --- |
| 1 | Ward X | Patient A | NOTT-113AA1 | EPI_ISL_573455 | 2020-09-25 | B.1.177.57 | 99.5887 |
| 1 | Ward X | Patient B | NOTT-113B35 | EPI_ISL_573460 | 2020-09-26 | B.1.177.57 | 99.5887 |
| 1 | Ward X | Patient C | NOTT-1134B2 | EPI_ISL_573420 | 2020-09-19 | B.1.177.57 | 98.8596 |
| 1 | Ward Y | Patient D | NOTT-113E1E |  | 2020-09-30 | B.1.177.57 | 75.0961 |
| 1 | Ward Y | Patient E | NOTT-114466 |  | 2020-10-07 |  | 0 |
| 1 | Ward Y | Patient E | NOTT-114536 | EPI_ISL_577172 | 2020-10-08 | B.1.177.57 | 99.5887 |
| 1 | Ward Y | Patient F | NOTT-114448 | EPI_ISL_577164 | 2020-10-07 | B.1.177.57 | 98.6021 |
| 1 | Ward Y | Patient G | NOTT-114545 |  | 2020-10-08 |  | 0 |
| 1 | Ward Y | Patient G | NOTT-1148C4 | EPI_ISL_584754 | 2020-10-08 | B.1.177.57 | 99.5954 |
| 1 | Ward Y | Patient H | NOTT-11493A | EPI_ISL_584756 | 2020-10-09 | B.1.177.57 | 99.5887 |
| 1 | Ward Y | Patient I | NOTT-1148A6 |  | 2020-10-08 |  | 14.5337 |
| 1 | Ward Y | Patient J | NOTT-1148D3 | EPI_ISL_584755 | 2020-10-08 | B.1.177.57 | 99.5887 |
| 1 | Ward Y | Patient K | NOTT-1151AA | EPI_ISL_584795 | 2020-10-11 | B.1.177.57 | 99.5954 |
| 1 | Ward Y | Patient L | NOTT-1151C8 | EPI_ISL_584797 | 2020-10-11 | B.1.177.57 | 99.5887 |
| 1 | Ward Y | Patient M | NOTT-114FDE | EPI_ISL_584786 | 2020-10-12 | B.1.177.57 | 98.8864 |
| 1 | Ward Y | Patient M | NOTT-115104 | EPI_ISL_584790 | 2020-10-13 | B.1.177.57 | 99.5887 |
| 1 | Ward Y | Patient N | NOTT-11582D | EPI_ISL_595268 | 2020-10-14 | B.1.177.57 | 96.084 |
| 1 | Ward Y | Patient O | NOTT-116B6F | EPI_ISL_595322 | 2020-10-17 | B.1.177.57 | 99.5887 |
| 1 | Ward J | Patient P | NOTT-117D95 | EPI_ISL_612590 | 2020-10-25 | B.1.177.57 | 99.582 |
| 1 | Ward J | Patient Q | NOTT-118945 | EPI_ISL_627685 | 2020-10-28 | B.1.177.57 | 99.582 |
| 1 | Ward J | Patient R | NOTT-1196D4 | EPI_ISL_627310 | 2020-11-01 | B.1.177.57 | 98.8663 |
| 1 | Ward J | Patient R | NOTT-1199CC | EPI_ISL_626811 | 2020-11-02 | B.1.177.57 | 96.9167 |
| 1 | Ward J | Patient S | NOTT-118E28 | EPI_ISL_627696 | 2020-10-29 | B.1.177.57 | 99.582 |
| 1 | Ward J | Patient T | NOTT-1186D5 | EPI_ISL_627309 | 2020-10-28 | B.1.177.57 | 99.5953 |
| 1 | Ward K | Patient U | NOTT-114F65 | EPI_ISL_584782 | 2020-10-12 | B.1.177.57 | 99.592 |
| 1 | Ward K | Patient V | NOTT-1151D7 | EPI_ISL_584798 | 2020-10-11 | B.1.177.57 | 97.8731 |
| 1 | Ward K | Patient V | NOTT-1181E3 |  | 2020-10-26 |  | 0 |
| 1 | Ward K | Patient V | NOTT-115359 | EPI_ISL_584805 | 2020-10-12 | B.1.177.57 | 99.582 |
| 1 | Ward K | Patient V | NOTT-11607F |  | 2020-10-18 |  | 0 |
| 1 | Ward K | Patient W | NOTT-11614F | EPI_ISL_595305 | 2020-10-18 | B.1.177.57 | 99.5887 |
| 1 | Ward K | Patient X | NOTT-11667D |  | 2020-10-20 |  | 0 |
| 1 | Ward K | Patient X | NOTT-1178B2 |  | 2020-10-23 |  | 0 |
| 1 | Ward K | Patient X | NOTT-117C5C | EPI_ISL_611582 | 2020-10-25 | B.1.177.57 | 95.8466 |
| 1 | Ward K | Patient X | NOTT-11667D |  | 2020-10-20 |  | 0 |
| 1 | Ward K | Patient X | NOTT-118A42 | EPI_ISL_627053 | 2020-10-28 | B.1.177.57 | 98.027 |
| 1 | Ward K | Patient Y | NOTT-11665F |  | 2020-10-20 |  | 0 |
| 1 | Ward K | Patient Y | NOTT-11767C | EPI_ISL_612578 | 2020-10-23 | B.1.177.57 | 99.582 |
| 1 | Ward K | Patient Y | NOTT-117C6B | EPI_ISL_612589 | 2020-10-25 | B.1.177.57 | 99.5887 |
| 1 | Ward K | Patient Y | NOTT-118BD6 |  | 2020-10-28 |  | 0 |
| 1 | Ward K | Patient Z | NOTT-11668C |  | 2020-10-20 |  | 0 |
| 1 | Ward K | Patient Z | NOTT-1178C1 |  | 2020-10-23 |  | 0 |
| 1 | Ward K | Patient Z | NOTT-117C4D | EPI_ISL_612588 | 2020-10-25 | B.1.177.57 | 99.582 |
| 1 | Ward K | Patient Z | NOTT-11AC3B |  | 2020-11-08 |  | 0 |
| 1 | Ward K | Patient Z | NOTT-11C13A |  | 2020-11-15 | B.1.177 | 62.0674 |
| 1 | Ward K | Patient Aa | NOTT-116CB7 |  | 2020-10-20 |  | 0 |
| 1 | Ward K | Patient Aa | NOTT-11768B | EPI_ISL_612579 | 2020-10-23 | B.1.177.57 | 99.5887 |
| 1 | Ward K | Patient Ab | NOTT-116E66 |  | 2020-10-20 |  | 0 |
| 1 | Ward K | Patient Ab | NOTT-117779 | EPI_ISL_612586 | 2020-10-23 | B.1.177.57 | 98.8463 |
| 1 | Ward K | Patient Ab | NOTT-116E57 |  | 2020-10-20 |  | 0 |
| 1 | Ward Y (STAFF) | Staff A | NOTT-114C04 | EPI_ISL_595234 | 2020-10-10 | B.1.177.57 | 98.5888 |
| 1 | Ward Y (STAFF) | Staff B | NOTT-114BAD |  | 2020-10-10 | B.1.1.138 | 89.6097 |
| 1 | Ward Y (STAFF) |  | NOTT-114C5F |  | 2020-10-10 |  | 0 |
| 1 | Ward Y (STAFF) | Staff C | NOTT-114BF8 | EPI_ISL_595233 | 2020-10-10 | B.1.177.57 | 99.5954 |
| 1 | Ward Y (STAFF) | Staff D | NOTT-114B70 | EPI_ISL_595232 | 2020-10-10 | B.1.177.16 | 99.5954 |
| 1 | Ward Y (STAFF) | Staff E | NOTT-114B61 | EPI_ISL_584764 | 2020-10-10 | B.1.177.57 | 99.5887 |
| 1 | Ward Y (STAFF) | Staff F | NOTT-114C40 | EPI_ISL_595235 | 2020-10-10 | B.1.177.57 | 99.582 |
| 1 | Ward Y (STAFF) | Staff G | NOTT-114D5C | EPI_ISL_638584 | 2020-10-11 | B.1.177.57 | 98.7928 |
| 1 | Ward Y (STAFF) | Staff H | NOTT-1152A7 |  | 2020-10-12 | B.1.177.57 | 79.8047 |
| 1 | Ward Y (STAFF) | Staff I | NOTT-115298 |  | 2020-10-12 | B.1.177.57 | 97.7996 |
| 1 | Ward Y (STAFF) | Staff J | NOTT-114F74 |  | 2020-10-12 | B.1.177.56 | 68.6365 |
| 1 | Ward Y (STAFF) | Staff K | NOTT-1163D0 | EPI_ISL_638585 | 2020-10-19 | B.1.177.57 | 99.5887 |
| 1 | Community | Community A | NOTT-113582 | EPI_ISL_549515 | 2020-09-20 | B.1.177.57 | 99.5887 |
| 1 | Community | Community B | NOTT-113810 | EPI_ISL_573434 | 2020-09-24 | B.1.177.57 | 99.5954 |
| 1 | Community | Community C | NOTT-113CAB | EPI_ISL_573471 | 2020-09-28 | B.1.177.57 | 99.5954 |
| 1 | Community | Community D | NOTT-113C9C | EPI_ISL_572582 | 2020-09-28 | B.1.177.57 | 97.7093 |
| 1 | Community | Community E | NOTT-11382F | EPI_ISL_573435 | 2020-09-24 | B.1.177.57 | 99.5887 |
| 1 | Hospital | Staff L | NOTT-115632 | EPI_ISL_595252 | 2020-10-15 | B.1.177.57 | 99.582 |
| 1 | Hospital | Bank Staff | NOTT-1153C2 | EPI_ISL_595240 | 2020-10-13 | B.1.177.57 | 99.5887 |
| 1 | Hospital | Patient – known contact | NOTT-11578A | EPI_ISL_595261 | 2020-10-14 | B.1.177.57 | 99.5954 |
| 1 | Hospital | Patient – known contact | NOTT-115131 | EPI_ISL_584792 | 2020-10-13 | B.1.177.57 | 99.5954 |
| 1 | Community | Untracked Community | ALDP-C3B98D | EPI_ISL_730926 | 2020-12-10 | B.1.177.57 | 96.3317 |
| 1 | Community | Untracked Community | QEUH-BF0D4D | EPI_ISL_709098 | 2020-12-03 | B.1.177.57 | 99.5954 |
| 1 | Community | Untracked Community | MILK-B38C6A | EPI_ISL_659059 | 2020-11-01 | B.1.177.57 | 99.5954 |
| 1 | Community | Untracked Community | CAMC-B3419C | EPI_ISL_659566 | 2020-11-05 | B.1.177.57 | 99.5954 |
| 1 | Community | Untracked Community | QEUH-B11B3D | EPI_ISL_642213 | 2020-11-02 | B.1.177.57 | 99.2042 |
| 1 | Community | Untracked Community | QEUH-AF068F | EPI_ISL_662767 | 2020-10-29 | B.1.177.57 | 99.5954 |
| 1 | Community | Untracked Community | MILK-ACD5CA | EPI_ISL_634428 | 2020-10-21 | B.1.177.57 | 99.5954 |
| 1 | Community | Untracked Community | QEUH-AD5592 | EPI_ISL_633879 | 2020-10-26 | B.1.177.57 | 99.5954 |
| 2 | Dialysis | Patient A | NOTT-113713 | EPI_ISL_573422 | 2020-09-21 | B.1.1.37 | 99.5954 |
| 2 | Dialysis | Patient B | NOTT-1137C8 | EPI_ISL_573430 | 2020-09-23 | B.1.1.37 | 99.5887 |
| 2 | Dialysis | Patient C | NOTT-113C23 | EPI_ISL_572706 | 2020-09-28 | B.1.177 | 98.6021 |
| 3 | Paediatric | Patient A | NOTT-1142F3 | EPI_ISL_577157 | 2020-10-07 | B.1.177.16 | 98.0236 |
| 3 | Paediatric | Staff A | NOTT-11469D | EPI_ISL_577187 | 2020-10-06 | B.1.36.17 | 99.5954 |

*^a^ Where genome completeness is greater than 90%, sequences are deposited in GISAID and accession IDs are provided. Genomes with ‘0’ completeness failed sequencing attempts. Pango Lineages are assigned using pangolin v 3.1.7 and pangolearn (*[*https://pangolin.cog-uk.io/*](https://pangolin.cog-uk.io/)*, 2021-07-09).*

*“Patient – known contact” describes individuals who were not part of the initial investigation but were known contacts of the cluster under investigation. Bank staff describes a case that was later linked to the outbreak.*

*Untracked Community samples are those which share the unique identifying SNP and lineage with cluster 1 but insufficient metadata are available to formally link them to the cluster. These samples were not sequenced as part of our local sequencing effort but are found through searching the wider COG-UK dataset.*
