# Supplementary material for: The Impact of Real-Time Whole-Genome Sequencing in Controlling Healthcare-Associated SARS-CoV-2 Outbreaks
Source: J Infect Dis. 2021 Sep 23;225(1):10–8. doi: 10.1093/infdis/jiab483 (PMC8522425; doi:10.1093/infdis/jiab483)
Supplement: jiab483_suppl_Supplementary_Table_S2 [file jiab483_suppl_supplementary_table_s2.docx]

**Supplementary Table 2** UK lineages within the NHS England Midlands (North Midland) Region as of 2020-12-15.

| UK Lineage ^a^ | Count | Global Lineage ^a^ | Count |
| --- | --- | --- | --- |
| UK1219 | 1668 | **B.1.177** | 1660 |
| UK1030 | 103 | **B.1.1** | 205 |
| UK5 | 99 | **D.1** | 103 |
| UK352 | 78 | **B.1.36.1** | 86 |
| UK2183 | 56 | **B.1.1.37** | 86 |
| UK2726 | 52 | **B.1.5** | 66 |
| UK2397 | 39 | **B.1** | 48 |
| UK1586 | 38 | **B.1.160** | 30 |
| UK1614 | 22 | **B.1.78** | 22 |
| UK1897 | 17 | **B.1.1.35** | 13 |
| UK3017 | 13 |  |  |
| UK649 | 12 |  |  |
| UK1736 | 11 |  |  |
| UK1805 | 11 |  |  |
| UK2815 | 10 |  |  |
| UK1780 | 9 |  |  |
| UK109 | 8 |  |  |
| UK1506 | 8 |  |  |
| UK1940 | 7 |  |  |
| UK3038 | 7 |  |  |

*^a^ Lineages determined by Pangolin COVID-19 Lineage Assigner (*[*https://pangolin.cog-uk.io/*](https://pangolin.cog-uk.io/)*).*
